# Mesenchymal stem cells promote ovarian reconstruction in mice

**DOI:** 10.1186/s13287-024-03718-z

**Published:** 2024-04-23

**Authors:** Jiazhao Li, Haonan Fan, Wei Liu, Jing Zhang, Yue Xiao, Yue Peng, Weijie Yang, Wenwen Liu, Yuanlin He, Lianju Qin, Xiang Ma, Jing Li

**Affiliations:** 1 State Key Laboratory of Reproductive Medicine and Offspring health, Nanjing Medical University, 210029 Nanjing, China; 2 Scientific Research Department, Wannan Medical College, 241002 Wuhu, China; 3 Center of Reproductive Medicine, The First Affiliated Hospital of Zhejiang University School of Medicine, 310003 Hangzhou, China; 4 Pathology Department, Nanjing Kingmed Medical Laboratory Co.,Ltd., 210032 Nanjing, China; 5 Assisted Reproduction Unit, Department of Obstetrics and Gynecology, Sir Run Run Shaw Hospital, Key Laboratory of Reproductive Dysfunction Management of Zhejiang Province, Zhejiang University School of Medicine, 310016 Hangzhou, China; 6 Women’s Hospital of Nanjing Medical University (Nanjing Maternity and Child Health Care Hospital), 21003 Nanjing, China; 7 State Key Laboratory of Reproductive Medicine and Offspring Health, Center of Clinical Reproductive Medicine, Jiangsu Province Hospital, The First Affiliated Hospital of Nanjing Medical University, 210029 Nanjing, China; 8 Prenatal Diagnosis Department, First Affiliated Hospital, Nanjing Medical University, 210029 Nanjing, China

**Keywords:** Mesenchymal stem cells, Fertility preservation, Artificial ovary

## Abstract

**Background:**

Studies have shown that chemotherapy and radiotherapy can cause premature ovarian failure and loss of fertility in female cancer patients. Ovarian cortex cryopreservation is a good choice to preserve female fertility before cancer treatment. Following the remission of the disease, the thawed ovarian tissue can be transplanted back and restore fertility of the patient. However, there is a risk to reintroduce cancer cells in the body and leads to the recurrence of cancer. Given the low success rate of current in vitro culture techniques for obtaining mature oocytes from primordial follicles, an artificial ovary with primordial follicles may be a good way to solve this problem.

**Methods:**

In the study, we established an artificial ovary model based on the participation of mesenchymal stem cells (MSCs) to evaluate the effect of MSCs on follicular development and oocyte maturation. P2.5 mouse ovaries were digested into single cell suspensions and mixed with bone marrow derived mesenchymal stem cells (BM-MSCs) at a 1:1 ratio. The reconstituted ovarian model was then generated by using phytohemagglutinin. The phenotype and mechanism studies were explored by follicle counting, immunohistochemistry, immunofluorescence, in vitro maturation (IVM), in vitro fertilization (IVF), real-time quantitative polymerase chain reaction (RT-PCR), and Terminal-deoxynucleotidyl transferase mediated nick end labeling(TUNEL) assay.

**Results:**

Our study found that the addition of BM-MSCs to the reconstituted ovary can enhance the survival of oocytes and promote the growth and development of follicles. After transplanting the reconstituted ovaries under kidney capsules of the recipient mice, we observed normal folliculogenesis and oocyte maturation. Interestingly, we found that BM-MSCs did not contribute to the formation of follicles in ovarian aggregation, nor did they undergo proliferation during follicle growth. Instead, the cells were found to be located around growing follicles in the reconstituted ovary. When theca cells were labeled with CYP17a1, we found some overlapped staining with green fluorescent protein(GFP)-labeled BM-MSCs. The results suggest that BM-MSCs may participate in directing the differentiation of theca layer in the reconstituted ovary.

**Conclusions:**

The presence of BM-MSCs in the artificial ovary was found to promote the survival of ovarian cells, as well as facilitate follicle formation and development. Since the cells didn’t proliferate in the reconstituted ovary, this discovery suggests a potential new and safe method for the application of MSCs in clinical fertility preservation by enhancing the success rate of cryo-thawed ovarian tissues after transplantation.

**Supplementary Information:**

The online version contains supplementary material available at 10.1186/s13287-024-03718-z.

## Background

With rapid advancements in cancer therapy, children and reproductive-age women are benefitting from overall improved survival rates [1]. It is well-documented that treatment of girls and women for cancer with radiation, chemotherapeutic drugs, or a combination of the two therapies can result in significant, and often irreversible, side-effect damage to the reproductive system [2, 3]. Anti-cancer therapy is often a cause of premature ovarian insufficiency (POI) due to the high sensitivity of the ovarian follicle reserve to chemotherapy and radiotherapy [4, 5]. Thus, there is an increased number of patients who received a gonadotoxic treatment and who later face fertility issues [6]. Overall, compared to the general population, women who undergo cancer treatment are 38% less likely to become pregnant. This reduction in the likelihood of subsequent pregnancies has been observed in nearly all types of cancer [7]. Consequently, the preservation of the ovarian reserve and prevention of infertility have become the primary quality of life concerns for patients and their physicians.

Fertility preservation refers to the use of surgical, pharmacologic, or laboratory techniques to provide assistance to women or men at risk of infertility in protecting and preserving their ability to have genetically derived offspring [8]. For women, common fertility preservation methods currently used include egg, embryo, and ovarian tissue freezing, whereas for unmarried or prepubertal women, freezing of the ovarian cortex is more appropriate [9–11]. After the patient’s condition has improved or resolved, the preserved ovarian cortex can be thawed and transplanted back to the patient. However, to minimize the risk of reintroducing cancer cells, an alternative approach involves culturing and developing the primordial follicles within the frozen cortex in vitro. This process can also incorporate biomaterials to construct an artificial ovary which can be transplanted into the patient’s body with the capability to produce mature eggs [6, 12]. Scientists have been trying to fully realize the in vitro culture and maturation of human follicles [13]. Telfer et al. applied a two-step culture method: ovarian tissue culture followed by follicle culture, and successfully obtained meiotic-capable eggs within a short period of time. However, further confirmation is still needed to determine whether these eggs have the ability to fertilize and support embryonic development [14].

Ovary, as a female reproductive organ, has a non-renewable nature. In order to delay ovarian aging or provide fertility preservation services for people with POI, artificial ovary has always been a difficult and hot spot of research in the field of reproduction and regenerative medicine [15, 16]. 3D-printed hydrogel scaffolds are being widely explored for the development of artificial ovaries that can support the growth and development of primordial, primary, and secondary follicles [17]. However, this approach does not address the fertility losses caused by issues such as gamete deficiency, impaired follicle formation, and development. To completely solve these problems, it is necessary to reconstruct the ovarian tissue at the single-cell level. The development and maturation of the oocyte is highly dependent on the follicular structure, which is therefore crucial in the process of artificial ovary construction [18]. Folliculogenesis is a long and complex process that involves a series of changes in the oocyte and its surrounding somatic cells [19]. Under physiological conditions, the primordial follicle is formed and enters a resting phase, providing an egg reserve for the entire reproductive cycle. Activation of this resting follicle and its entry into the growth and development phase is dependent on local signals originating from the ovary [20, 21]. It has been shown that only ovarian somatic cells at a specific developmental period have the ability to form follicles. However, when these cells are reconstituted with germ cells to create a recombinant ovary, a significant proportion of oocytes undergo apoptosis. Moreover, due to the disruption of the internal regulatory mechanisms, the resulting follicles experience accelerated activation and development which ultimately results in the loss of the ovary’s function very soon after the reorganization [22]. In order to achieve the complete three-dimensional reconstruction of ovarian tissue, how to form follicular structures with high efficiency and ensure the normal development of follicles becomes the primary problem to be solved.

Mesenchymal stem cells (MSCs) are a class of multipotential adult stem cells with the ability to differentiate directionally into adipose, bone, cartilage, and other cell types [23]. It has been the most widely studied cell type in the field of regenerative medicine because of its easy accessibility, self-replication, directed differentiation, and low immunogenicity [24, 25]. The results from animal models have been successfully applied in clinical practice. One example is the transplantation of bone marrow mesenchymal stem cells (MSCs) from patients to repair damaged endometrium, which can restore normal uterine function during conception [26]. Studies investigated the use of MSCs to enhance ovarian function in mice with premature ovarian failure, showing a significant improvement in the internal environment of the ovaries [27–31]. While these studies have highlighted the potential of MSCs in restoring and protecting ovarian function, most researchers believe that this is achieved through the regulation of factors secreted by MSCs. The differentiation of MSCs into intra ovarian cells has not been extensively explored and it requires further investigation to better understand the mechanisms involved in the reconstruction of ovarian function. In this study, we established an artificial ovary model by mixing newborn ovarian single cells with MSCs at a 1:1 ratio. This artificial ovary model improved the survival rate of oocytes to form primordial follicles and then the following follicular development and oocyte maturation. Mesenchymal stem cells may be a potential cell resource in remodeling follicular structure for fertility preservation in future.

## Materials and methods

### Experimental animals

Mice were obtained from Vital River Laboratories (Beijing, China) and housed in the animal facility at Nanjing Medical University. Mice were maintained under a 12/12-h dark-light cycle at 22 °C with free access to food and water. All animal protocols were approved by the Committee on the Ethics of Animal Experiments at Nanjing Medical University. Ovaries of P2.5 ICR females were used for reconstituted ovaries and transplanted into kidney capsules of the same strain of female mice at 8–10 weeks of age. Control oocytes for IVF were collected from P23 ICR female mice by superovulation and adult male B6D2F1 mice (10–14 weeks old) were chosen as sperm donors. Female ICR mice at 2- to 3-week-old were used for isolation of MSCs. All efforts were made to minimize the number and suffering of the animals used in the study. A total of 250 mice were used. The number of mice that contributed data for analysis in each experiment was indicated in figure legends. All animal experiments adhere to the ARRIVE guidelines.

### Isolation and culture of MSCs from mouse compact bone

MSCs were isolated as previously described [32]. Briefly, 2- to 3-week-old female mice were sacrificed by cervical dislocation. Rinse the animal liberally in a beaker with 100 ml of 70%(vol/vol) ethanol for 3 min. Place the mouse in a 100-mm sterile glass dish and incise the skin, disassociate the muscles, sever the femurs below the femoral head, and disconnect hindlimbs from the trunk. Dissect the humeri by excising the forelimb at the axillaries. Pull the skin down. Clean the muscles and tendons from the humeri, tibiae, and femurs. Place the bones on sterile gauze and then carefully rub them to remove the attached soft tissue from the bone. Before further processing, store the bones in a 35-mm sterile glass dish with 5 ml minimum essential medium α (α-MEM)supplemented with 0.1% (vol/vol) penicillin/streptomycin and FBS 2% (vol/vol). Insert a 0.45-mm syringe needle into the bone cavity and flush marrow out with 3 ml of α-MEM. Wash the bone cavities thoroughly at least three times using a syringe until the bones become pale. For MSCs GFP labeling, the virus was stored and used in strict accordance with the instructions(GPLVX-CMV-ZsGreen1). Viral transfection for labeling GFP was performed on ICR mouse MSCs cultured to P4 generation. The virus was added when the cells were cultured to 50% fusion, and after 48 h of infection, the medium was changed to normal cell culture medium. After 48 h of culture, the expression of green fluorescent protein in the cells was observed by fluorescence microscopy.

### Flow cytometric analysis and multilineage differentiation

Cells cultured to P5 generation were harvested for flow characterization of MSC-specific markers. Trypsin-digested cells were washed twice with phosphate buffer saline (PBS) (4-8 °C) and then suspended in cold PBS at a concentration of 100 µl of 1×10^6^ cells per EP tube and incubated with PE-conjugated anti-mouse CD29, CD31, CD34, CD44, Sca-1 antibodies and incubate the cells at 4 °C for 30 min, protected from light. To identify cellular activity, PI was added and the cells were incubated for 15 min at 4 °C, protected from light. After the antibody incubation, the cells were washed twice with cold PBS and resuspended with fresh PBS, and the sample and preparation were completed. The prepared samples were analyzed by analytical flow cytometry to detect the expression of each antibody in the cells and mouse MSCs differentiation analysis was performed with Passage 5 mouse MSCs in osteogenic (MUXUC-90021, Caygen, USA), adipogenic (MUXUC-90031, Caygen, USA) or chondrogenic differentiation medium (MUXUC-9004, Caygen, USA) and differentiated cells were identified by Alizarin Red, Oil O Red, and Alcian Blue.

### Aggregation of ovaries with mouse bone marrow mesenchymal stem cells

P2.5 newborn ovaries were harvested by carefully removing oviducts and ovarian bursa in L-15 medium containing 3 mg/mL bovine serum albumin ( BSA)/PBS (0.01 M, pH 7.4, Hyclone, USA). The ovaries were further digested in 1mL PBS supplemented with 0.25% trypsin, 1 mM ethylenediaminetetracetic acid (EDTA), and incubated at 37 °C for 6 min with gentle agitation every two minutes. Then centrifuge at 3000 g and stop at maximum speed. Aspirate the trypsin from the EP tube and add 1mL collagenase type IV,0.01% DNase I and incubated at 37 °C for 9 min with gentle agitation every three minutes. To stop the digestion, 10% fetal bovine serum (FBS) was added and the cell suspensions were centrifuged at 3000g and stopped at maximum speed at 4 °C. The digestion solution was removed, and cells were suspended by adding α-MEM with 50 µg/ml phytohemagglutinin (L1668, Sigma-Aldric) and incubated at 37 °C for 10 min followed by centrifugation at 3000gfor 10 s to aggregate the cells. The tubes were then rotated 180° and centrifuged again for approximately 30s. This double centrifugation protocol we used was modified from a previous study [22].

### In vitro culture and grafting of reconstituted ovaries

Reconstituted ovaries (rOvaries) were gently removed from the tubes and cultured on inserts (PICM03050, Millipore, USA) with 1.5 ml culture media added in the bottom of each well. The culture medium was α-MEM supplemented with 0.23 mM pyruvic acid, 50 mg/l streptomycin sulfate, 75 mg/l penicillin G, 0.03U/ml FSH and 3 mg/ml BSA. Ovarian cells were reconstituted as controls, and MSCs were added to reconstitute them as treatments, and grouped for culture. Reconstructed ovaries were cultured overnight in a 37 °C incubator at 100% humidity and 5% CO2 awaiting transplantation. The samples were collected after 24, 48 and 96 h of culture and fixed in neutral formalin and paraformaldehyde (PFA) to detect apoptosis and other conditions. For long-term culture, the culture medium was changed every other day and a half. For tissue transplantation, the aggregated artificial ovaries were surgically implanted beneath the renal capsules of bilaterally ovariectomized host females, and the same receptor mice with the left side as the treatment group and the right side as the control group. Some animals were sacrificed at 14 days after transplantation to assess follicular development, some animals were sacrificed at 18 days to collect GV oocytes for IVM, and some animals were injected with 5 IU human chorionic gonadotrophin(hCG), (Sansheng Bio Tech, China) at 24 days to collect MII mature oocytes 12 h later for IVF randomly.

### IVM and IVF

In IVM studies, germinal vesicle (GV) oocytes were obtained from grafted MSC-rOvaries and cultured in M2 medium (Sigma, USA) at 37 °C and 5% CO2 under mineral oil (Sigma, USA) conditions. Following 16 h of culture, the ratio of MII mature oocytes/GV oocytes was assessed and MII oocytes were collected for morphological and immunofluorescence examination. For in vivo studies, recipient mice transplanted for 24 days were given a single injection of 5 IU hCG to induced ovulation, and the transplanted MSC-rOvaries were collected into M2 medium containing 0.1% hyaluronidase (H3506, Sigma, USA) 14 h later. MII oocytes were harvested directly by mechanical puncture with a fine needle. The control GV and MII oocytes were collected from P25 ICR mice after injection of pregnant mare serum gonadotropin (PMSG) and hCG for superovulation. At the time of the IVF study, donor sperm was collected from B6D2F1 male mice, which were capacitated by incubation in human tubal fluid medium (HTF) (MR-070-D, Millipore, USA) in oil for 1 h at 37 °C, 5% CO2. The MII oocytes were then incubated in 250 µl of medium containing spermatozoa (2-3 ×10^5^/ml) for 6-8 h. Upon fertilization, zygotic cells with clear pronuclei were transferred into fresh HTF medium overnight until the 2-cell embryonic stage. Then 2-cell embryos were cultured in small droplet KSOM medium (MR-020P-5 F, Millipore, USA) until blastocyst stage. Embryo development was assessed as the ratio of 2-cell embryos to zygotes and the ratio of blastocysts to zygotes.

## Real-time PCR

The total RNA of the rOvaries was isolated using Trizol Reagent (Invitrogen, USA) according to the method provided by the manufacturer. rOvaries were measured for RNA concentration using a spectrophotometer (NanoDrop 2000c, Thermo Scientific, USA). 500 ng of RNA/reaction from each sample was reverse transcribed using the FastQuant RT Kit (Tianyuan Biotechnology, China) to produce cDNA. cDNA was then analyzed on an ABI Step One Plus platform (Thermo Scientific, USA) using a SYBR-Green mix (Applied Biological Materials, Canada) on the ABI Step One Plus platform (Thermo Scientific, USA) for real-time PCR analysis, using the actin amplification signal as an internal control. The specificity of the PCR products was assessed by melting curve analysis and the amplicon size was determined by 2% agarose gel electrophoresis.

### Immunohistochemistry

Reconstituted ovaries were collected and fixed in 4% buffered formalin for paraffin embedding and sectioning. To detect the expression of AMH and PCNA, 5 μm sections were deparaffinized and rehydrated, and endogenous peroxidase activity was blocked by incubation in 3% hydrogen peroxide in methanol for 15 min. The sections were then boiled in 0.01 M citrate buffer to retrieve the antigen. After blocking by goat serum (ZSGB-Bio, China) for 1 h, primary antibodies were incubated overnight at 4 °C, and 3,3’-Diaminobenzidine (DAB) reagent was used for coloration on the second day. Non-immune IgGs were applied as negative controls.

### Follicle counting

The reconstituted ovaries from operated mice were collected and fixed in 10% buffered formalin overnight for serial sectionst(5 μm) and hematoxylin and eosin staining. To evaluate follicular development in operated mice, all follicles were counted at every fifth section using the fractionator and nucleator principles [33]. All sections were counted by two independent individuals for comparison.

### Immunofluorescence

MII Oocytes from IVM and superovulated mice were collected and the cumulus cells were removed by M2 medium containing 0.1% hyaluronidase. Oocytes were fixed in 4% paraformaldehyde for 30 min and then permeabilized with 0.5% Triton X-100 (Sigma-Aldrich, USA) for 20 min at room temperature. After blocking in 1% BSA/PBS solution for 1 h, oocytes were incubated with anti-β-tubulin antibody overnight at 4℃. After washing in 1% BSA/PBS, oocytes were incubated with Alexa Fluor 488 goat anti-rabbit secondary antibody (Invitrogen, Carlsbad, USA) for 40 min at room temperature. The nuclei were then counterstained with 0.01 mg/ml Hoechst 33342 (Invitrogen, USA) for 15 min. All the oocytes were put on the slides and observed by Confocal (Zeiss, LSM700, Germany). For rOvaries, immunofluorescence was performed with antibodies for VASA (Abcam Ab13840, 1: 400), PCNA (CST, catalog no.13110,1: 16,000), After incubation overnight at 4 °C, the primary antibodies were washed out and sections were incubated with relative secondary antibodies at RT for 1 h. The secondary antibodies include Alexa Fluor 488 donkey anti-rabbit (Invitrogen, USA, catalog no. A21206; 1: 500), Alexa Fluor 594 donkey anti-mouse (Invitrogen, catalog no. A21203, 1: 500), Alexa Fluor 488 goat anti-mouse secondary antibodies(Invitrogen, catalog no. A21202, 1: 500). Then the nuclei were stained with 0.01 mg/ml Hoechst 33342 (Invitrogen, catalog no. H1339) for 20 min and sections were viewed under a laser scanning confocal microscope(Zeiss, LSM700, Germany).

### TUNEL assays

TUNEL assays were performed on 5 μm sections with the TUNEL Apoptosis Detection Kit (Alexa Fluor 640)( Yeasen, catalog. no 40308) according to the manufacturer’s instructions.

### Immunoblotting analysis

Proteins were extracted by RIPA lysis buffer (P0013B, Beyotime Institute of Biotechnology) containing protease inhibitor cocktails (M221, Amresco). A total of 10-30 µg proteins in each sample were loaded and separated by electrophoresis (165-8000, Bio-Rad, USA). After electronic transfer (170-3930, Bio-Rad, USA), the PVDF membranes (88250, Thermo Fisher, USA) were blocked in 5% skimmed milk-TBST (TBS containing 0.1% Tween 20) for 30 min and incubated overnight at 4 °C with primary antibodies. After washing with TBST (5 ml) for three times, the HRP-conjugated relative secondary antibodies were then used to detect proteins through enhanced chemiluminescence (RPN2232, GE Healthcare, Washington, NY) on the Tanon 5200 analysis system.

### Statistical analysis

The software of GraphPad Prism 5.0 and SPSS 20.0 were used to do the chi-square test, or one-way ANOVA and Mann-Whitney U-test to evaluate differences between groups. Data are showed as mean ± SEM. *P* < 0.05 was considered to be statistically significant.

## Results

### Reconstruction of ovarian function with mouse BM-MSCs and ovarian cells

**Fig. 1:**
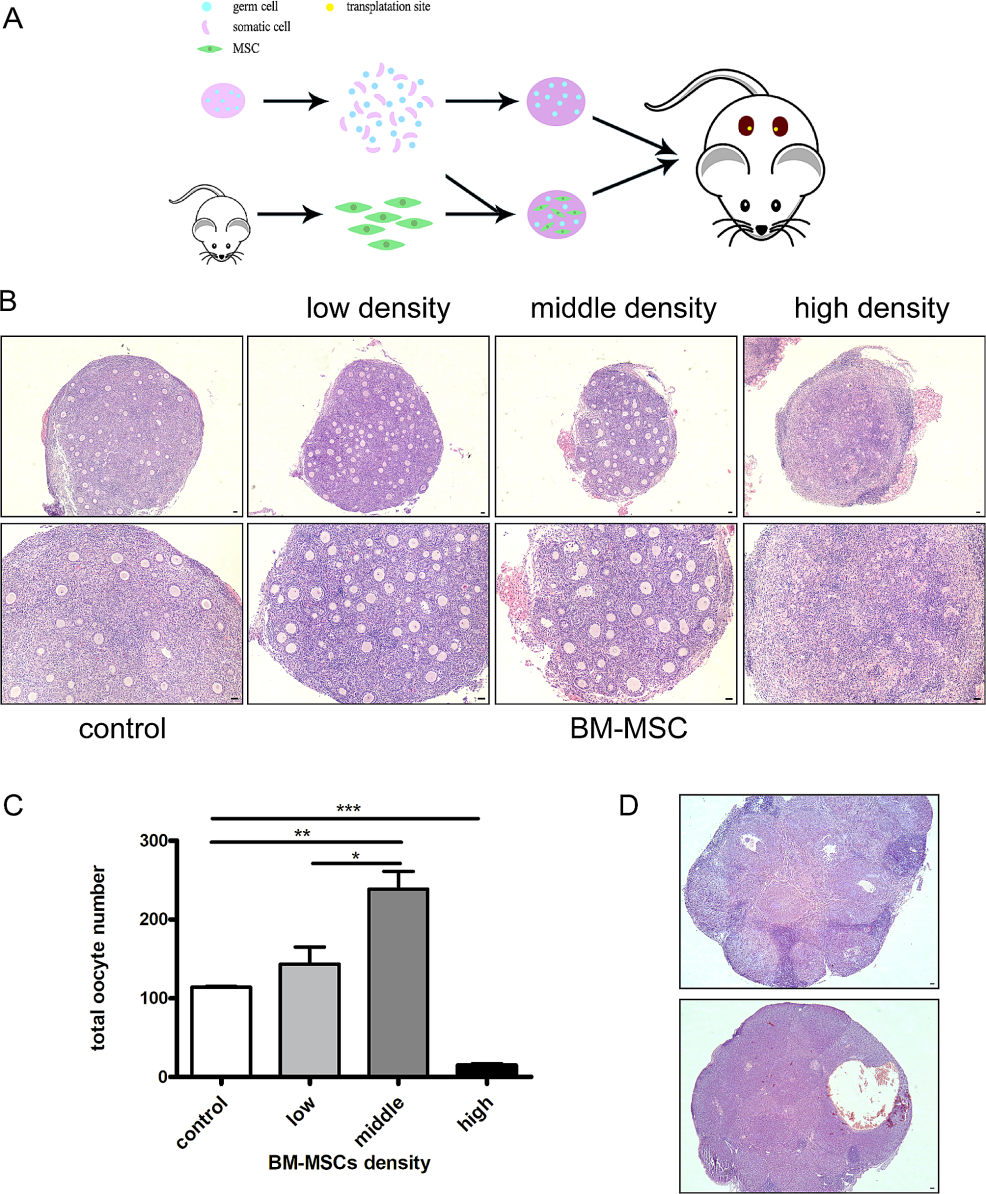
Reconstruction of ovarian function with mouse mesenchymal stem cells and ovarian cells. (**A**) Scheme for reconstitution procedure. (**B**)HE staining of different density of MSCs in reaggregated ovaries after transplanted 14 days. (**C**)Numbers of oocyte in reaggregated ovaries with different densities of MSCs. *n* = 5/per group. (**D**) Long-term observation of intra-tissue morphology in reconstituted ovaries with MSCs. HE staining of reaggregated ovaries with MSCs after transplantation 4 weeks and 6 weeks. Data are shown as mean ± SEM. *p<0.05. **p<0.01 ***p < 0.001. Scale bars,200 μm.

To see the effects of MSCs on the reconstruction of ovarian tissue, mouse BM-MSCs were isolated and passaged from mouse compact bone (Figure S1A). They were positive for the mesenchymal stem cell markers CD29, CD44, and the progenitor cell marker Sca-1 but were negative for the hematopoietic marker CD45 and the endothelial cell marker CD31 (Figure S1B). Moreover, these cells could differentiate into adipocytes, chondrocytes, and osteoblasts after in vitro induction (Figure S1D-F). In mouse ovaries, follicle assembly starts at E17.5 (embryonic day 17.5), and primordial follicle formation is nearly completed by P2.5 (postnatal day 2.5) [34, 35]. Next, we collected P2.5 ovaries and dissociated them into single cells. The aggregation of ovaries were obtained from ovarian cells with phytohemagglutinin. After transplanting beneath the renal capsules of bilaterally ovariectomized host female mouse, follicular structure formed and continued to develop in these aggregations. Based on this, we introduced different concentrations of BM-MSCs into the aggregated system (Fig. 1A). We divided the MSC-reconstituted ovaries into a low concentration group, a medium concentration group, and a high concentration group according to the amount of MSCs added, and the ratios of MSCs to ovarian cells were 1 to 2, 1 to 1, and 2 to 1, respectively. We found that by adding different concentrations of MSCs in the reconstituted ovaries, follicular development was significantly different after transplanting these reconstituted ovaries to the recipient mouse kidney capsule for 14 days. The results of H&E staining (Fig. 1B) and oocyte counting (Fig. 1C) showed that the addition of mouse MSCs at medium concentrations could promote follicle formation and follicular development. However, in reconstituted ovaries with high concentrations of mouse MSCs, exogenous cells hinder the formation and development of follicles in the aggregates. The reason may be that too many exogenous cells dispersed ovarian cells so that broke up the communications and interactions of ovarian cells which are essential for ovarian follicle assembly.

### Safety evaluation after BM-MSCs being incorporated in the reconstituted ovary

As we know, the medical application of stem cells refuses safety risks. In order to evaluate the safety of BM-MSCs participated in ovarian reconstruction, we transplanted reconstructed ovaries for long-term tracking and obtained samples at 4 weeks, 6 weeks, and 8 weeks after transplantation to observe whether there were tumors in reconstituted ovaries. After completing histological analysis of ovarian samples from all transplant recipient mice, we found no tumors or other adverse conditions. The transplanted tissue was absorbed and formed a small white spot-like shape under the renal capsule after 8 weeks of transplantation. Moreover, H&E staining of ovarian tissue after 4 weeks and 6 weeks of transplantation showed lots of corpus luteum on the ovarian section, suggesting normal ovulation occurred during follicular development (Fig. 1D). The results indicate the safety for the application of MSCs in ovarian reconstruction.

**Mouse BM-MSCs benefit ovarian development in reconstituted ovaries.**

**Fig. 2:**
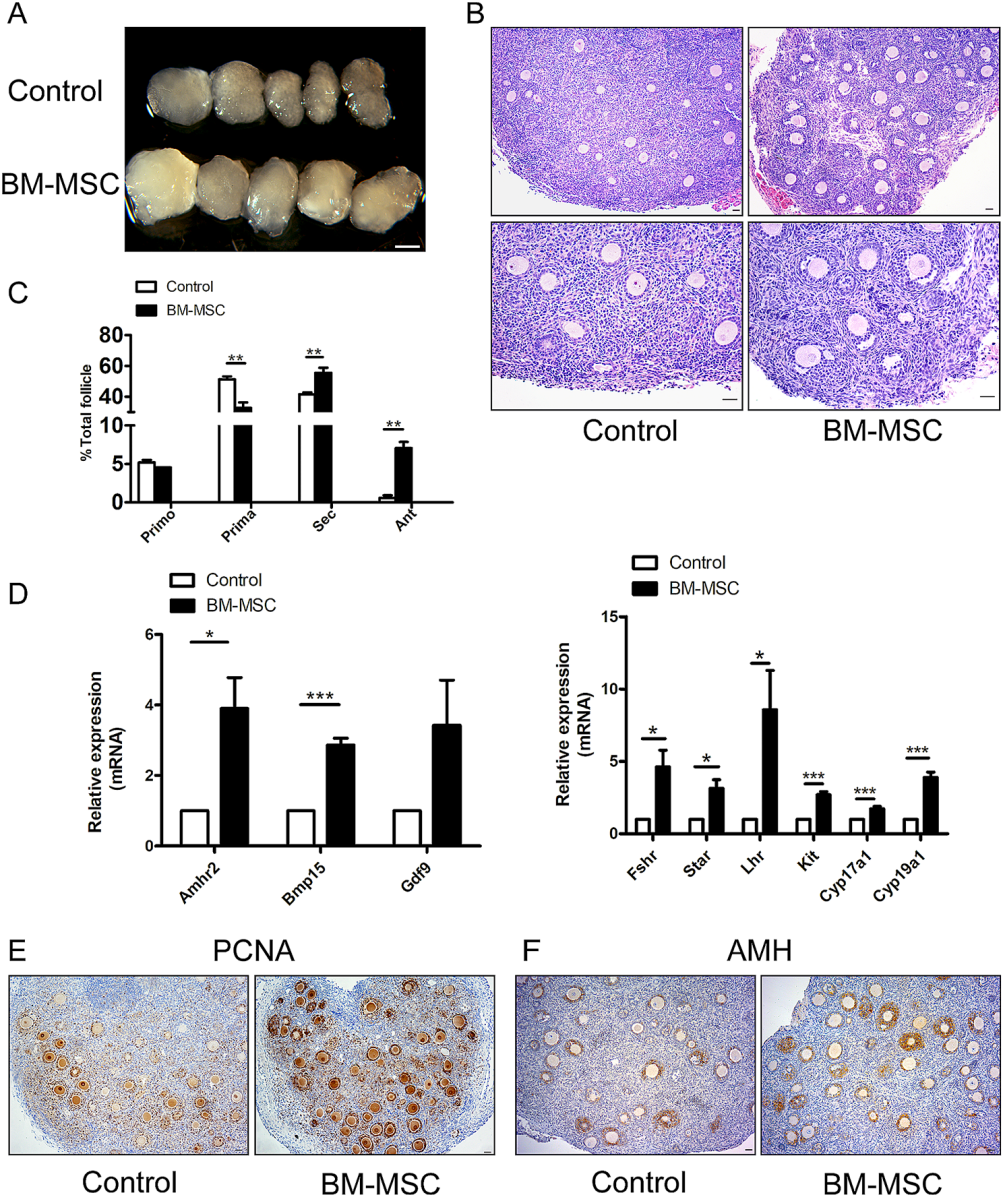
Mouse MSCs benefit ovarian development in reconstituted ovaries. Reconstituted ovaries were obtained after 14 days of in vivo development. (**A**) Morphology of control-rOvaries and MSC-rOvaries at 14 days after transplantation. Scale bars, 1 mm. (**B**) HE staining was performed to observe the internal growth of the reconstituted ovaries, and it was seen that the follicular development in the MSC-rOvaries group was better than that of the control group. Scale bar,50 μm. (**C**) Distribution of follicles in r-Ovaries without and with MSCs. *n* = 3/per group. Primo, primordial follicle; Prima, primary follicle; Sec, secondary follicle; Ant, antral follicle. Scale bar, 50 μm. (**D**) Relative expression of follicular growth and development related gene in the rOvaries were assessed by real-time PCR. *n* = 3/per group. Data are presented as means ± SEM.*P < 0.05 and ***p < 0.001. (**E**) Immunohistochemistry analyses the expression of PCNA in reaggregated ovaries after transplanted 14 days. Scale bar, 50 μm. (**F**) Immunohistochemistry analyses expression of AMH in aggregated ovaries after transplanted 14 days. Scale bar, 50 μm.

To further evaluate the effects of MSCs on follicular growth and development in reconstituted ovaries, we used the best ratio of ovarian cells and BM-MSCs as 1:1 to reconstruct ovaries. After 24 h of in vitro culture, the reconstructed ovaries with or without BM-MSCs were transplanted under the renal capsule of the same ovariectomized adult recipient mouse in pairs, and the samples were collected after 14 days of transplantation. The collected ovaries were shown as Fig. 2A and the volume of MSC-ovaries was greater than that of control-ovaries. Histological analysis further revealed accelerated follicular development with more large antral follicles being observed in the MSC-ovaries (Fig. 2B). After counting follicles by means of serial sections, the results showed that the proportion of secondary and antral follicles in total follicles of MSC-ovaries was higher than that in the control group (Fig. 2C). RT-PCR results demonstrated the increased expression of oocyte development genes, *Bmp15, Gdf9* and *Kit* and follicle growth-related genes *Amhr2, Fshr*, *Star*, *Lhr*, *Cyp17a1* and *Cyp19a1* (Fig. 2D). Proliferating cell nuclear antigen (PCNA) staining showed the stronger signals in the BM-MSCs reconstituted ovaries (Fig. 2E). Immunohistochemistry with AMH also revealed more secondary follicles and antral follicles (Fig. 2F). These results show that mesenchymal stem cells can promote the survival and development of follicles.

### The aggregated ovaries with mouse BM-MSCs achieved normal oocytes maturation

**Fig. 3:**
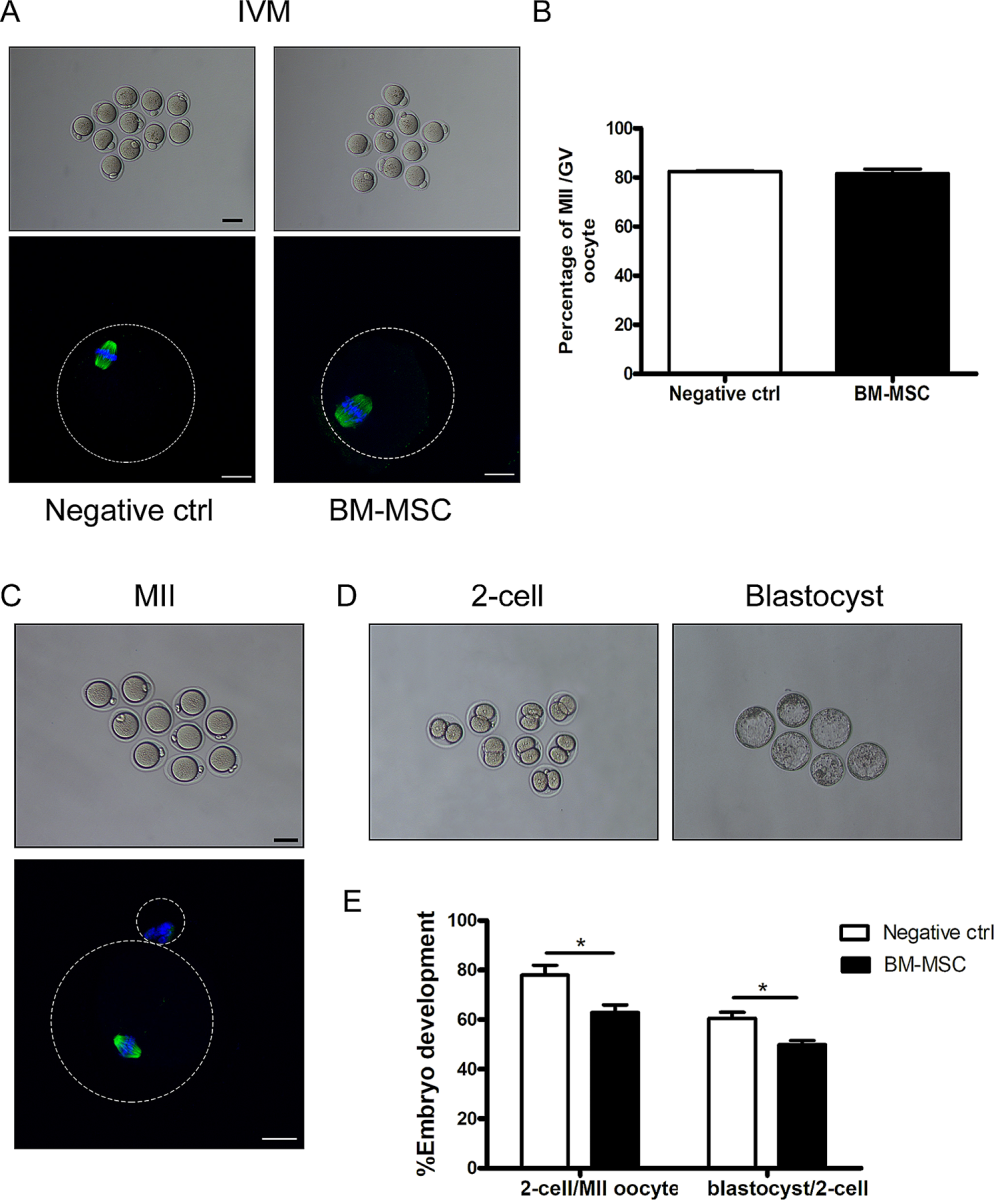
Evaluation of oocyte quality by IVM, IVF and early embryonic development. Aggregated ovaries with mouse MSCs were transplanted into the kidney capsules of recipient mice for 18 days. GV oocytes for IVM were collected by directly puncturing fully grown follicles under the microscope and mature MII oocytes were retrieved after a single injection of hCG into recipient mice after transplanted 24 days (IVO). (**A**) Morphology of MII oocytes after IVM. Oocytes from superovulated ovaries of 3-week-old mice were used as negative controls (Negative ctrl). And Immunofluorescence of β-tubulin on spindle of MII oocytes. Green, β-tubulin; Blue, Nuclear staining with Hoechst 33342. (**B**) Percentages of MII oocytes with aberrant spindles after IVM treatment. (**C**) Morphology of MII oocytes obtained from MSC-rOvaries after a single injection of hCG into recipient mice after transplanted 24 days (IVO). And Immunofluorescence of β-tubulin on spindle of MII oocytes. Green, β-tubulin; Blue, Nuclear staining with Hoechst 33342. (**D**)Representative 2-cell embryos and blastocysts after IVO oocytes being fertilized in vitro (IVF). (**E**) Efficiency of early embryonic development. Percentage of IVO mature oocytes in negative controls and MSC-rOvaries group capable of developing into 2-cell embryos and blastocysts. All experiments were performed with at least three replicates. Data were shown as mean ± SEM. *p < 0.05. Scale bar, 50 μm.

To evaluate the developmental potential of follicles and oocytes after BM-MSCs being incorporated into the aggregated ovary, germinal vesicle oocytes were collected from reconstituted ovaries for IVM after transplantation for 18 days. Since it was difficult to develop the aggregated ovaries without BM-MSCs at the same stage, GV oocytes from normal female ICR mice were used as negative controls. The morphology of MII oocytes was shown by β-tubulin staining (Fig. 3A) and no difference on the oocyte maturation rate was observed between negative control and reconstituted ovary groups (Fig. 3B). We also collected mature oocytes from BM-MSCs reconstituted ovaries after transplantation for 24 days with a single injection of hCG. Mature oocytes from normal 24-day ICR mice after superovulation were served as normal controls. As shown in Fig. 3C, MII oocytes obtained from reconstituted ovaries have normal morphology and immunofluorescence of β-tubulin revealed normal spindle distributions in mature oocytes. After in vitro fertilization using donor sperm, mature oocytes from reconstituted ovaries developed from 2-cell embryos to blastocysts after 96 h of culture, but the 2-cell rate (63% vs. 78%) and the blastocyst rate (49% vs. 60%) obtained from oocytes in reconstituted group were lower than those from normal ICR mice (Fig. 3D and E). The above results show that BM-MSCs reconstituted ovary can achieve the entire process of follicular development and produce normal functional oocytes.

### Mouse mesenchymal stem cells reduced apoptosis in reconstituted ovaries

**Fig. 4:**
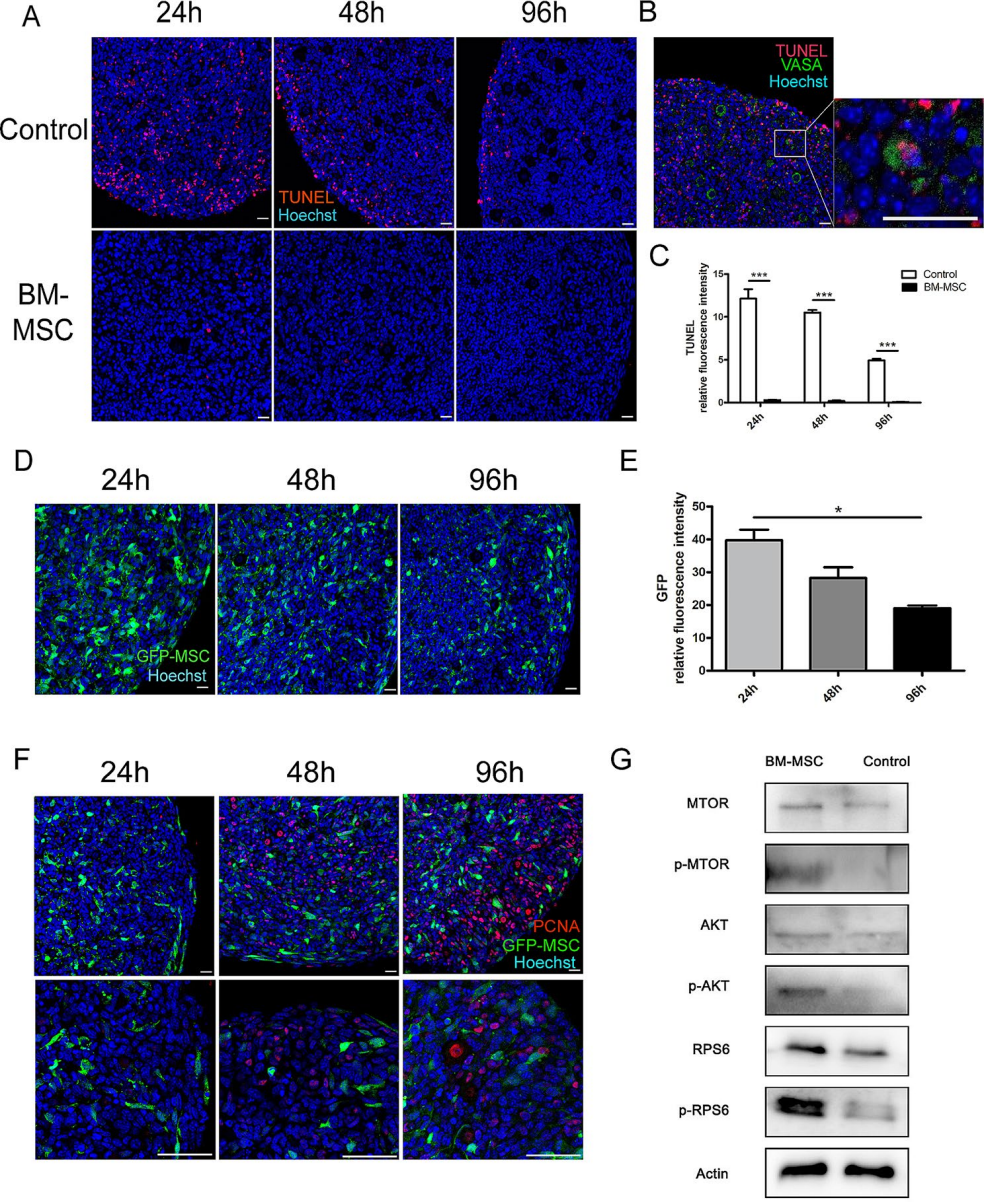
Mouse mesenchymal stem cells reduce apoptosis in reconstituted ovaries. (**A**) TUNEL assay of reaggregated ovaries after in vitro culture. (**B**) The Control group of rOvaries cultured in vitro for 24 h, TUNEL co-stained with immunofluorescence of germ cell marker VASA antibody. The apoptotic signal was present in VASA-positive oocytes. (Scale bar: 50 μm.). (**C**) The statistical analysis of TUNEL was performed to detect apoptosis within the tissues of rOvaries at various time points in the early post-recombinational period. ***p < 0.001. *n* = 4/each group. GFP-labeled MSCs were used to track the growth of reaggregated ovaries. (**D**) GFP-MSCs in reaggregated ovaries after recombination. (**E**) Analysis of scanning intensity statistics of green fluorescent signals carried by mesenchymal stem cells in MSC-rOvaries. *n* = 5/each group. *p<0.05. (**F**) Immunofluorescence labeling of proliferation by PCNA antibody, PCNA red fluorescent signal did not co-localize with GFP. (**G**) Western blot showed the increased phosphorylation of Akt, mTOR and RPS6 in BM-MSCs reconstituted ovaries after 96 h of culture.Scale bar, 50 μm.

Next, we used the TUNEL assay to assess apoptosis in reconstituted ovaries with or without BM-MSCs and the aggregated ovaries were collected after 24, 48, and 96 h of culture. As shown in Fig. 4A and B, the reconstitution of ovary induced a lot of apoptosis in both somatic cells and oocytes (TUNEL^+^, VASA^+^). The highest cellular apoptosis (∼12%) was observed after 24 h of aggregation and then the apoptosis rate decreased gradually with less than 5% apoptotic cells in aggregated ovaries after 96 h of culture (Fig. 4C). However, nearly no apoptotic signals were found in BM-MSCs reconstituted ovary even at the 24-hour time point when a lot of apoptosis was observed in the control group. Thus, adding BM-MSCs promoted the survival of ovarian cells and avoided a large loss of oocytes shortly after reconstitution.

### Dynamic tracking of mouse MSCs during the growth of reconstituted ovaries

Mesenchymal stem cells have a strong ability to self-replicate in the process of in vitro culture, but this uncontrolled intensity of proliferation is risky for in vivo applications [25]. We reconstituted ovaries with green fluorescent protein (GFP)-labeled BM-MSCs (Figure S1B, 83% of GFP positive cells) and followed them to observe the proliferation and location of these cells involved in reconstituted ovaries. There was a clear downward trend in the density of green fluorescent cells from the first 24 h to 96 h after reconstitution (Fig. 4D). Statistical analysis of green fluorescence intensity further verified the result (Fig. 4E). We then labeled proliferating cells with PCNA staining and we found the PCNA signals did not coincide with green fluorescence labeled MSCs. This means that MSCs didn’t proliferate in the aggregated ovary, but the numbers of oocytes and ovarian somatic cells exhibited a gradual increase within the reconstituted ovary. (Fig. 4F). BM-MSCs function to maintain oocyte survival and accelerate the proliferation of ovarian somatic cells during the assembly of follicles. Western blot then revealed the increased phosphorylation of Akt, mTOR and RPS6 in BM-MSCs reconstitute ovaries after 96 h of culture. It suggests BM-MSCs may promote follicle formation and follicle activation through the activation of PI3K/mTOR signaling pathway (Fig. 4G).

**Fig. 5:**
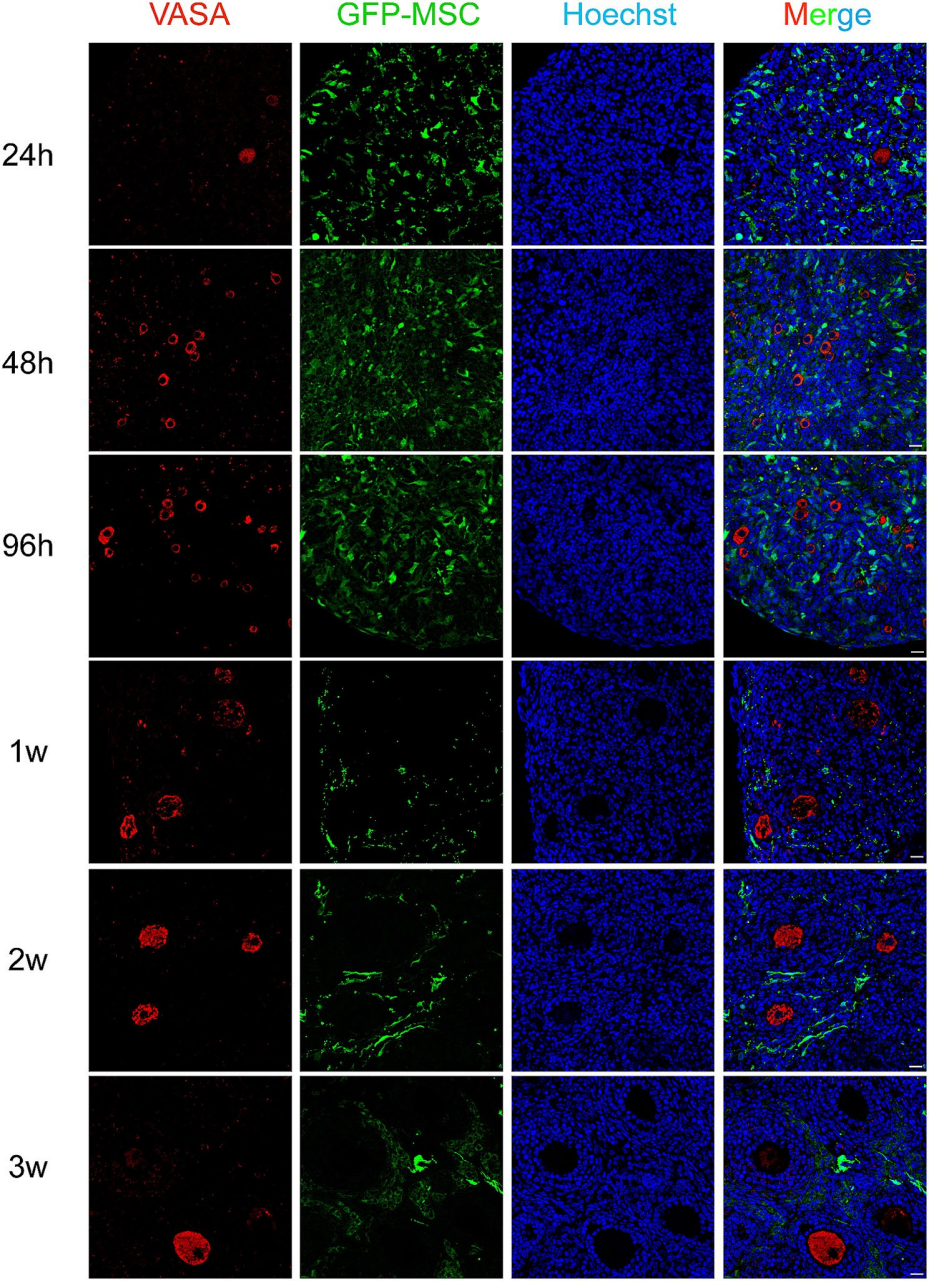
Dynamic tracking of green fluorescent protein-labeled MSCs in recombinant ovaries from 24 h to 3weeks after aggregation. Scale bar, 50 μm.

**Fig. 6:**
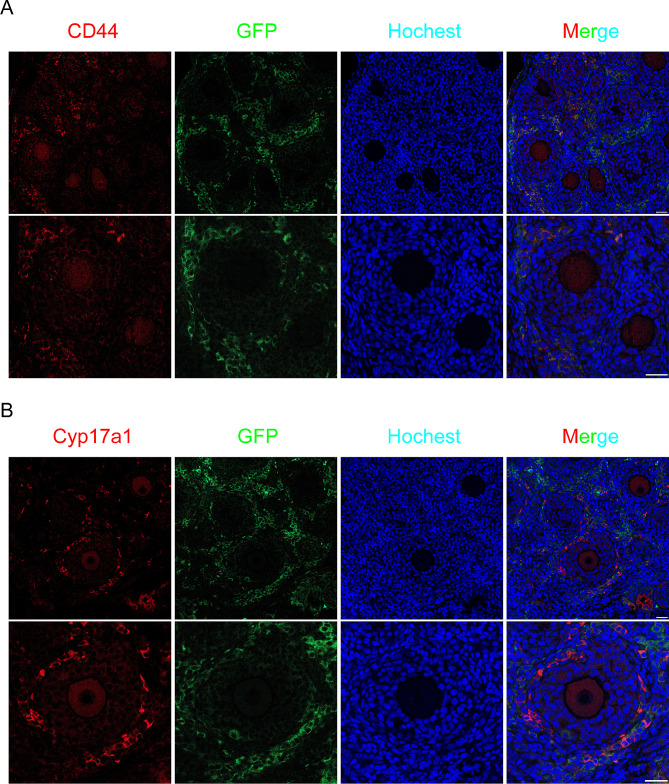
Immunofluorescence staining of MSC cell marker CD44 (**A**) and follicular theca cell marker Cyp17a1(**B**) in green fluorescent protein-labeled MSC-rOvaries. Scale bar: 50 μm.

To further explore how MSCs behaved and contributed in reconstituted ovaries, GFP labeled BM-MSCs were tracked in the reconstitution of the ovary and oocytes were labeled with VASA staining. In the first 96 h of ovarian reconstruction, the MSCs distributed evenly with ovarian cells, however, they didn’t involve in the reassembly of follicles. After aggregated ovaries being transplanted into recipient mice, follicular development was observed and we found MSCs mainly distributed in the stroma of reconstituted ovaries around growing follicles. Together, we also noticed a significant decrease of MSC cell numbers and fluorescence in the reconstituted ovary (Fig. 5). To see if the MSCs participate in the differentiation of theca cells, we first stained the reconstituted ovary with the MSC cell marker CD44 after transplantation for two weeks. While the majority of CD44-positive cells also exhibited GFP fluorescence, we observed a subset of cells that exclusively expressed GFP (Fig. 6A). Theca cells were then labeled with CYP17a1 and we found although in most cases, GFP and CYP17a1 positive cells were not colocalized with each other, double positive cells were still observed in the theca layer (Fig. 6B). Together, our result revealed the pivotal role of BM-MSCs in the restoration of the ovarian function. These cells aid in the survival of aggregated ovarian tissues during the first several days of reconstruction. Although they don’t proliferate or contribute to the formation of follicle structure, their presence around growing follicles indicates their involvement in the differentiation of the theca layer.

## Discussion

In this study, we incorporated BM-MSCs into ovarian cells to reconstitute the ovarian structure by using phytohemagglutinin. Although previous studies have demonstrated promising results regarding the ability of MSCs to facilitate the regeneration of bone, blood vessels, skin, peripheral nerves, and alleviate symptoms of local ischemia in various organs like the heart, kidneys, and brain, it is important to note that MSCs have been sometimes viewed as a panacea within the field of tissue repair and regenerative medicine [24, 36]. Studies also showed that co-cultured of MSCs with follicles could promote follicle survival and development [30, 31, 37]. In the study, when we tried to reconstitute the ovarian structure with different concentrations of BM-MSCs, we found overloading BM-MSCs hindered the interaction between oocytes and ovarian somatic cells and inhibited follicle formation and development. Therefore, only when BM-MSCs and ovarian cells were mixed at a ratio 1:1, the most promoting effect was found in the reconstituted ovary.

When ovarian reconstitution was performed with the optimal number of MSCs, we found that the involvement of MSCs promoted the development of the reconstituted ovaries. First, at the initial stage of ovarian reconstitution, the incorporation of MSCs reduced or even avoided a large loss of ovarian cells including both oocytes and ovarian somatic cells caused by the preparation of ovarian aggregates. Existing studies have shown that MSCs have a significant role in repairing damage and inhibiting apoptosis. They create a repair microenvironment by releasing transforming growth factor-beta 1(TGF-β1) after tissue injury and reduce apoptosis, inflammatory response, and immune response at the site of injury [24, 38, 39]. Therefore, MSCs have a reparative effect in the reconstituted ovary. Secondly, by utilizing kidney capsule transplantation of recombinant ovaries, we observed that the presence of MSCs resulted in enhanced follicle formation and development compared to ovaries without MSCs. This enhancement was evident through various evaluations, including morphological analysis of follicular growth, follicle counting, PCNA staining, as well as labeling of secondary and antral follicles with AMH. The activation of the PI3K/mTOR signaling pathway and the results of the expression of oocyte development-related genes, such as *Bmp15*, *Gdf9* and *Kit*, and the expression of hormone production-related genes in the recombinant ovaries further verified the above conclusions. It has been shown that MSCs can increase the expression of transforming growth factor-beta (TGF-β) family members in follicles during co-culture with MSCs [37]. We know that the TGF-β family including Gdf9 and Bmp15 plays an important role in the development of preantral follicles [20, 40, 41]. This confirms the experimental results we obtained that follicle development in MSCs reconstituted ovaries was better than in the control group.

To evaluate whether the artificial recombinant ovary, which was constructed with the participation of BM-MSCs, has normal folliculogenesis and oocyte maturation. Oocytes at GV stage were removed from the recombinant ovary and matured in vitro (IVM), and mature MII oocytes were also collected for IVF to evaluate embryonic development from two-cell embryos to blastocysts. These results showed that the recombinant ovary with BM-MSCs could complete normal follicular development and oocyte maturation. In other words, this recombinant ovary model with MSCs we established is a new method to constructing artificial ovaries for rebuilding normal ovarian function. It is well known that the expansion and improvement of fertility preservation methods for female cancer patients have consistently posed challenges and remained as prominent areas of research in the field of reproduction. Artificial ovaries and various follicle culture methods have particularly garnered significant attention in this context [6, 16, 42].. Among them, 3D-printed artificial ovaries consisted of primordial, primary, secondary follicles and 3D-printed hydrogel scaffolds have gained wide attention [17]. However, such artificial ovary based on follicle units cannot solve the problem of fertility loss due to folliculogenesis disorders or gamete deficiency. The regeneration of reproductive organs by inducing pluripotent stem cells and embryonic stem cells to generate gametes in female mice have been reported [43]. And with this cell-based artificial ovary model, our study demonstrated adult stem cells can also be used for constructing artificial ovary with stem cell-induced artificial eggs.

Through dynamic tracking of BM-MSCs in reconstituted ovaries by GFP labeled MSCs, we found that MSCs didn’t proliferate in the reconstituted ovary and they didn’t participate in the formation of follicles after reconstitution. In the recombinant ovary, BM-MSCs accompanied the growth and development of the follicle, and their distribution gradually gathered around the periphery of the growing follicle. Previous studies have demonstrated that the follicular theca layer consists of theca endocrine cells located internally, and an outer layer of fibrous connective tissue derived from fibroblast-like cells. The theca exterior also contains vascular tissue, immune cells, and stroma [44].

Therefore, the follicular theca layer serves crucial roles in maintaining the structural integrity of the follicle, as well as providing nutrients to the granulosa cell layer and oocytes. Furthermore, the theca cells are responsible for producing important endocrine regulators such as androgens (testosterone and dihydrotestosterone), as well as growth regulators like morphogenetic proteins (BMPs) and TGF-β. These regulatory molecules are essential for facilitating follicular growth and development [45–47]. In the study, although most GFP positive cells are expressed MSC marker CD44, we still found some theca cells showed GFP and CYP17a1 double positive signals. This means the potential that BM-MSCS can differentiate into theca cells, however, more experiments are needed to explore this conjecture. In the study, we also concerned about the safety of MSCs in ovarian recombination, and we did not find the proliferation of MSCs and no tumors and other undesirable conditions was observed after a long-term tracking. Therefore, we conclude that MSCs have a good application prospect for constructing artificial ovaries.

## Conclusions

In summary, we established a reconstituted ovary model by using newborn ovarian cells including oocytes and somatic cells with BM-MSCs. We found the addition of BM-MSCs increased the survival rate of oocytes in the recombinant ovary and promoted follicular growth and development. The MSC recombinant ovary has normal folliculogenesis and oocyte development and maturation. During the growth and development of the recombinant ovary, BM-MSCs gradually spread around the growing follicles. After immunostaining with theca cell marker CYP17a1, some theca cells showed double positive signals with GFP fluorescence of BM-MSCs. Whether they can differentiate into theca cells remain to be studied further. Our results reveal MSCs may be a good resource to reconstruct ovarian tissue in female fertility preservation.

### Electronic supplementary material

Below is the link to the electronic supplementary material.

Supplementary Material 1

Supplementary Material 2

## Data Availability

Data sharing is not applicable to this article as no datasets were generated during the current study. The images depicted in Figs. 1-6 and S1 are drawn by ourselves.

## Abbreviations

MSCs: Mesenchymal stem cells
BM-MSCs: Bone marrow derived mesenchymal stem cells
IVM: In vitro maturation
IVF: In vitro fertilization
RT-PCR: Real-time quantitative polymerase chain reaction
TUNEL: Terminal-deoxynucleotidyl transferase mediated nick end labeling
POF: Premature ovarian failure
E2: 17β-estradiol
AMH: Anti-Mullerian hormone
FSH: Follicle-stimulating hormone
HGF: Hepatocyte growth factor
VEGF: Vascular endothelial growth factor
IGF: Insulin like growth factor
GFP: Green fluorescent proteinn
HE: Hematoxylin-eosin
PCNA: proliferating cell nuclear antigen
Bmp15: Bone morphogenetic protein 15
Gdf9: Growth differentiation factor 9
Amhr2: Anti-Mullerian hormone receptor 2
Fshr: Follicle-stimulating hormone receptor
Star: Steroidogenic acute regulatory protein
Lhr: Luteinizing hormone receptor
Cyp17a1: Cytochrome P450, family 17, subfamily A, polypeptide 1
Cyp19a1: Cytochrome P450, family 19, subfamily A, polypeptide 1
GV: Germinal vesicle
TGF-β1: Transforming growth factor-betae1
α-MEM: Minimum essential medium α
PBS: Phosphate buffer saline9
BSA: Bovine serum albumin
EDTA: Ethylenediaminetetracetic acid
FBS: Fetal bovine serum
PFA: Paraformaldehyde
HCG: Human chorionic gonadotrophinm
PMSG: Pregnant mare serum gonadotropin
HTF: Human tubal fluid medium
DAB: 3,3’-Diaminobenzidine.

## References

[1] Campbell SB, Woodard TL (2020). An update on fertility preservation strategies for women with cancer. Gynecol Oncol.

[2] Akahori T, Woods DC, Tilly JL (2019). Female Fertility Preservation through Stem Cell-based ovarian tissue reconstitution in Vitro and ovarian regeneration in vivo. Clin Med Insights Reproductive Health.

[3] Kim H, Kim H, Ku SY (2018). Fertility preservation in pediatric and young adult female cancer patients. Annals Pediatr Endocrinol Metabolism.

[4] Balachandren N, Davies M (2017). Fertility, ovarian reserve and cancer. Maturitas.

[5] Roness H, Kashi O, Meirow D (2016). Prevention of chemotherapy-induced ovarian damage. Fertil Steril.

[6] Donnez J, Dolmans MM (2017). Fertility preservation in women. N Engl J Med.

[7] Anderson RA, Brewster DH, Wood R, Nowell S, Fischbacher C, Kelsey TW, Wallace WHB (2018). The impact of cancer on subsequent chance of pregnancy: a population-based analysis. Hum Reprod.

[8] Medrano JV, Andres MDM, Garcia S, Herraiz S, Vilanova-Perez T, Goossens E, Pellicer A (2018). Basic and Clinical Approaches for Fertility Preservation and Restoration in Cancer patients. Trends Biotechnol.

[9] Smitz J, Dolmans MM, Donnez J, Fortune JE, Hovatta O, Jewgenow K, Picton HM, Plancha C, Shea LD, Stouffer RL (2010). Current achievements and future research directions in ovarian tissue culture, in vitro follicle development and transplantation: implications for fertility preservation. Hum Reprod Update.

[10] Rodriguez-Wallberg KA, Anastacio A, Vonheim E, Deen S, Malmros J, Borgstrom B (2020). Fertility preservation for young adults, adolescents, and children with cancer. Ups J Med Sci.

[11] Hong YH, Park C, Paik H, Lee KH, Lee JR, Han W, Park S, Chung S, Kim HJ (2023). Fertility Preservation in Young Women with breast Cancer: a review. J Breast cancer.

[12] Saito S, Yamada M, Yano R, Takahashi K, Ebara A, Sakanaka H, Matsumoto M, Ishimaru T, Utsuno H, Matsuzawa Y (2023). Fertility preservation after gonadotoxic treatments for cancer and autoimmune diseases. J Ovarian Res.

[13] Pors SE, Ramlose M, Nikiforov D, Lundsgaard K, Cheng J, Andersen CY, Kristensen SG (2019). Initial steps in reconstruction of the human ovary: survival of pre-antral stage follicles in a decellularized human ovarian scaffold. Hum Reprod.

[14] McLaughlin M, Albertini DF, Wallace WHB, Anderson RA, Telfer EE (2018). Metaphase II oocytes from human unilaminar follicles grown in a multi-step culture system. Mol Hum Reprod.

[15] Cho E, Kim YY, Noh K, Ku SY (2019). A new possibility in fertility preservation: the artificial ovary. J Tissue Eng Regen Med.

[16] Salama M, Woodruff TK (2019). From bench to bedside: current developments and future possibilities of artificial human ovary to restore fertility. Acta Obstet Gynecol Scand.

[17] Laronda MM, Rutz AL, Xiao S, Whelan KA, Duncan FE, Roth EW, Woodruff TK, Shah RN (2017). A bioprosthetic ovary created using 3D printed microporous scaffolds restores ovarian function in sterilized mice. Nat Commun.

[18] West ER, Shea LD, Woodruff TK (2007). Engineering the follicle microenvironment. Semin Reprod Med.

[19] Jones ASK, Shikanov A (2019). Follicle development as an orchestrated signaling network in a 3D organoid. J Biol Eng.

[20] McGee EA, Hsueh AJ (2000). Initial and cyclic recruitment of ovarian follicles. Endocr Rev.

[21] Del Valle JS, Chuva de Sousa Lopes SM. Bioengineered 3D ovarian models as Paramount Technology for Female Health Management and Reproduction. Bioengineering 2023, 10(7). 10.3390/bioengineering10070832 PMC10376580 37508859

[22] Eppig JJ, Wigglesworth K, Pendola FL (2002). The mammalian oocyte orchestrates the rate of ovarian follicular development. Proc Natl Acad Sci USA.

[23] Uccelli A, Moretta L, Pistoia V (2008). Mesenchymal stem cells in health and disease. Nat Rev Immunol.

[24] Caplan AI, Correa D (2011). The MSC: an injury drugstore. Cell Stem Cell.

[25] Volarevic V, Bojic S, Nurkovic J, Volarevic A, Ljujic B, Arsenijevic N, Lako M, Stojkovic M (2014). Stem cells as new agents for the treatment of infertility: current and future perspectives and challenges. Biomed Res Int.

[26] Ding L, Li X, Sun H, Su J, Lin N, Peault B, Song T, Yang J, Dai J, Hu Y (2014). Transplantation of bone marrow mesenchymal stem cells on collagen scaffolds for the functional regeneration of injured rat uterus. Biomaterials.

[27] Li J, Mao Q, He J, She H, Zhang Z, Yin C (2017). Human umbilical cord mesenchymal stem cells improve the reserve function of perimenopausal ovary via a paracrine mechanism. Stem Cell Res Ther.

[28] Tomaszewski CE, Constance E, Lemke MM, Zhou H, Padmanabhan V, Arnold KB, Shikanov A (2019). Adipose-derived stem cell-secreted factors promote early stage follicle development in a biomimetic matrix. Biomaterials Sci.

[29] Xia X, Yin T, Yan J, Yan L, Jin C, Lu C, Wang T, Zhu X, Zhi X, Wang J (2015). Mesenchymal stem cells enhance angiogenesis and follicle survival in human cryopreserved ovarian cortex transplantation. Cell Transpl.

[30] Guo C, Ma Y, Situ Y, Liu L, Luo G, Li H, Ma W, Sun L, Wang W, Weng Q (2023). Mesenchymal stem cells therapy improves ovarian function in premature ovarian failure: a systematic review and meta-analysis based on preclinical studies. Front Endocrinol.

[31] Huang Y, Zhu M, Liu Z, Hu R, Li F, Song Y, Geng Y, Ma W, Song K, Zhang M (2022). Bone marrow mesenchymal stem cells in premature ovarian failure: mechanisms and prospects. Front Immunol.

[32] Zhu H, Guo ZK, Jiang XX, Li H, Wang XY, Yao HY, Zhang Y, Mao N (2010). A protocol for isolation and culture of mesenchymal stem cells from mouse compact bone. Nat Protoc.

[33] Flaws JA, Abbud R, Mann RJ, Nilson JH, Hirshfield AN (1997). Chronically elevated luteinizing hormone depletes primordial follicles in the mouse ovary. Biol Reprod.

[34] He Y, Chen Q, Dai J, Cui Y, Zhang C, Wen X, Li J, Xiao Y, Peng X, Liu M (2021). Single-cell RNA-Seq reveals a highly coordinated transcriptional program in mouse germ cells during primordial follicle formation. Aging Cell.

[35] Li J, Kawamura K, Cheng Y, Liu S, Klein C, Liu S, Duan EK, Hsueh AJ (2010). Activation of dormant ovarian follicles to generate mature eggs. Proc Natl Acad Sci USA.

[36] Maldonado VV, Patel NH, Smith EE, Barnes CL, Gustafson MP, Rao RR, Samsonraj RM (2023). Clinical utility of mesenchymal stem/stromal cells in regenerative medicine and cellular therapy. J Biol Eng.

[37] Xia X, Wang T, Yin T, Yan L, Yan J, Lu C, Zhao L, Li M, Zhang Y, Jin H (2015). Mesenchymal stem cells facilitate in Vitro Development of Human Preantral follicle. Reproductive Sci.

[38] Caplan AI, Dennis JE (2006). Mesenchymal stem cells as trophic mediators. J Cell Biochem.

[39] Kurpinski K, Lam H, Chu J, Wang A, Kim A, Tsay E, Agrawal S, Schaffer DV, Li S (2010). Transforming growth factor-beta and notch signaling mediate stem cell differentiation into smooth muscle cells. Stem Cells.

[40] Otsuka F, Yao Z, Lee T, Yamamoto S, Erickson GF, Shimasaki S (2000). Bone morphogenetic protein-15. Identification of target cells and biological functions. J Biol Chem.

[41] Hreinsson JG, Scott JE, Rasmussen C, Swahn ML, Hsueh AJ, Hovatta O (2002). Growth differentiation factor-9 promotes the growth, development, and survival of human ovarian follicles in organ culture. J Clin Endocrinol Metab.

[42] Oktay K, Harvey BE, Loren AW. Fertility preservation in patients with Cancer: ASCO Clinical Practice Guideline Update Summary. J Oncol Pract 2018:JOP1800160. 10.1200/JOP.18.00160 29768110

[43] Hikabe O, Hamazaki N, Nagamatsu G, Obata Y, Hirao Y, Hamada N, Shimamoto S, Imamura T, Nakashima K, Saitou M (2016). Reconstitution in vitro of the entire cycle of the mouse female germ line. Nature.

[44] Richards JS, Ren YA, Candelaria N, Adams JE, Rajkovic A (2018). Ovarian Follicular Theca Cell Recruitment, differentiation, and impact on fertility: 2017 update. Endocr Rev.

[45] Lee S, Kang DW, Hudgins-Spivey S, Krust A, Lee EY, Koo Y, Cheon Y, Gye MC, Chambon P, Ko C (2009). Theca-specific estrogen receptor-alpha knockout mice lose fertility prematurely. Endocrinology.

[46] Liu C, Peng J, Matzuk MM, Yao HH (2015). Lineage specification of ovarian theca cells requires multicellular interactions via oocyte and granulosa cells. Nat Commun.

[47] Young JM, McNeilly AS (2010). Theca: the forgotten cell of the ovarian follicle. Reproduction.

