# Supplementary figures and images for: Mesenchymal stem cells promote ovarian reconstruction in mice
Source: Stem Cell Res Ther. 2024 Apr 23;15:115. doi: 10.1186/s13287-024-03718-z (PMC11036642; doi:10.1186/s13287-024-03718-z)

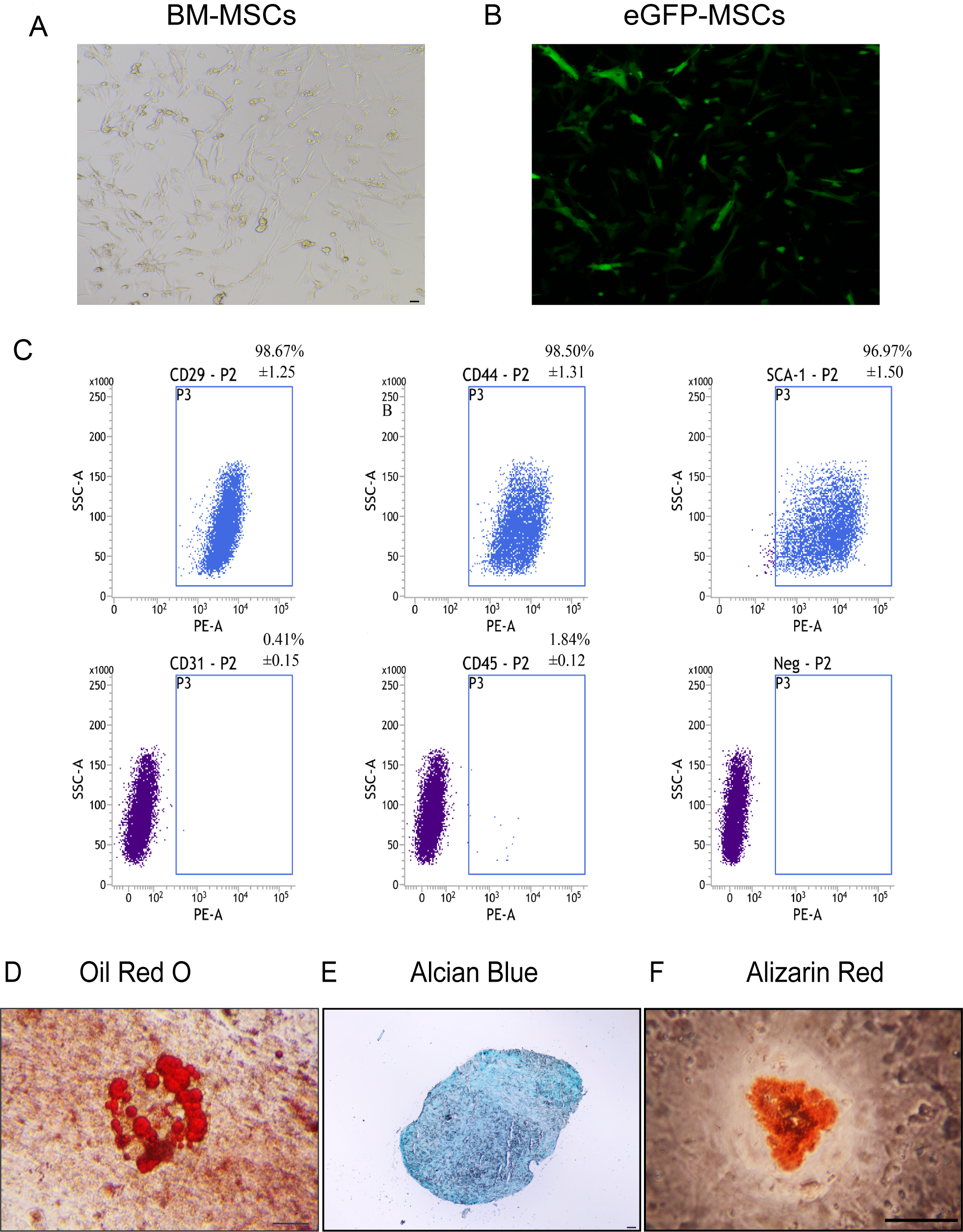

Supplement: Supplementary file 1 — Supplementary Material 1 [file 13287_2024_3718_MOESM1_ESM.tif]
