# Supplementary material for: Mesenchymal stem cells promote ovarian reconstruction in mice
Source: Stem Cell Res Ther. 2024 Apr 23;15:115. doi: 10.1186/s13287-024-03718-z (PMC11036642; doi:10.1186/s13287-024-03718-z)
Supplement: Supplementary file 2 — Supplementary Material 2 [file 13287_2024_3718_MOESM2_ESM.docx]

**Figure S1. Characterization of mouse BM-MSCs.** (A) Morphology of 3rd passage mouse BM-MSCs. (B) MSCs were green labeled with GFP. (C) Flow cytometry analysis of immune markers of mouse mesenchymal stem cells. FACS results showed that these cells were homogenously positive for mesenchymal markers CD29, CD44; progenitor cells markers Sca-1 but negative for hematopoietic markers CD45; endothelial cells markers CD31. (D) Adipogenesis of MSCs was stained with oil-red-O. (E) Chondrocytic cells differentiated from MSCs were identified with Alcian blue staining. (F) Osteoblastogenesis of MSCs was assayed with alizarin red staining. MSCs had the potential to differentiate to multiple lineages. All experiments were performed with at least three replicates. Scale bars: 50μm.
